# Newborn Health Interventions and Challenges for Implementation in Nepal

**DOI:** 10.3389/fpubh.2016.00015

**Published:** 2016-02-11

**Authors:** Resham Bahadur Khatri, Shiva Raj Mishra, Vishnu Khanal, Khageshwor Gelal, Subas Neupane

**Affiliations:** ^1^Saving Newborn Lives Program, Save the Children, Kathmandu, Nepal; ^2^Nepal Development Society, Kathmandu, Nepal; ^3^School of Public Health, Curtin University, Perth, Australia; ^4^Child Health Division, Ministry of Health and Population, Kathmandu, Nepal; ^5^School of Health Sciences, University of Tampere, Tampere, Finland

**Keywords:** interventions, challenges, Nepal, neonatal mortality rate, health systems

## Abstract

Neonatal mortality is a major challenge in reducing child mortality rates in Nepal. Despite efforts by the Government of Nepal, data from the last three demographic and health surveys show a rise in the contribution of neonatal deaths to infant and child mortality. The Government of Nepal has implemented community-based programs that were piloted and then scaled up based on lessons learned. These programs include, but are not limited to ensuring safe motherhood, birth preparedness package, community-based newborn care package, and integrated management of childhood illnesses. Despite the implementation of such programs on a larger scale, their effective coverage is yet to be achieved. Health system challenges included an inadequate policy environment, funding gaps, inadequate procurement, and insufficient supplies of commodities, while human resource management has been found to be impeding service delivery. Such bottlenecks at policy, institutional and service delivery level need to be addressed incorporating health information in decision-making as well as working in partnership with communities to facilitate the utilization of available services.

## Introduction

Disparity in the unequal distribution of the neonatal mortality rate (NMR) has been reported among different regions, religious, and ethnic groups in Nepal. There are higher rates of NMR among poor socio-economic groups, Muslims and Dalits, as well as people living in remote areas such as the mid and far-western regions of the country (1–3). Neonates born to older mothers and those with high parity have been reported to be at more than four times higher risk of neonatal death (3). Similarly, newborns of illiterate mothers were twice as likely to suffer neonatal death than those born to women with higher levels of education (1). Importantly, having four antenatal care (ANC) visits or the childbirth assisted by a skilled birth attendant (SBA) halves the risk of neonatal death. However, utilization of such services is very low in high-risk sub-groups of women (3, 4). In response to these issues, many programs and newborn health interventions are being implemented but encounter various health system challenges. This study aims to summarize the health system challenges and possible solutions for specific interventions focused on reducing neonatal deaths.

## Trends of Child Mortality in Nepal

Nepal has been successful in achieving millenium development goal (MDG) 4 of reducing the under-five mortality rate (U5MR) by two-thirds from the level in 1990 (2, 5). During the past 20 years, the nation has witnessed unequal progress in the reduction of mortalities among children; with a 54% reduction in U5MR, a 55% reduction in post-neonatal mortality rate (PNMR) but with a slower reduction in neonatal mortality rate (NMR) of 34%. The U5MR declined by 7.6%/year, while the NMR declined by only 2.6%/year over the period 2000–2010 (6). During the period of 2006–2011, the proportion of NMR in the U5MR and infant mortality rate (IMR) increased from 42 to 61% and 63 to 72%, respectively, with no change in NMR, which remained the same (33/1000 live births) in both Nepal Demographic and Health Surveys (NDHS) (7).

**Figure 1:**
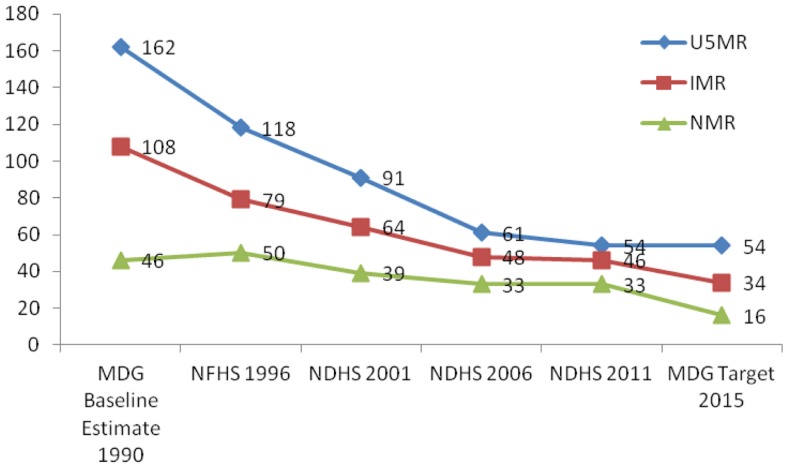
**Neonatal mortality trends in Nepal (Per 1000 live births)**. Source: MDG Baseline Estimate 1990, NFHS 1996, NDHS 2001, NDHS 2006, and NDHS 2011.

The U5MR and IMR decreased rapidly from 1996 to 2006, but the rate of reduction slowed between 2006 and 2011 due to constant NMR (7). The target of MDG 4 to reduce U5MR to 54/1000 live births has already been achieved, but reducing IMR to 34/1000 live births and NMR to 16/1000 live births by the end of 2015 has been difficult, although formal statistics are yet to be released (2). Figure 1 shows the trend of mortality rates among the under-fives, infants, and neonates over the last 20 years (2).

Further analysis of the 2011 NDHS showed that about two in five neonatal deaths occur on the first day after birth with more than 8 out of 10 neonatal deaths occurring in the first week of life (3). Furthermore, the high proportion of stillbirths indicates poor quality of care during the intra-partum period (8). The verbal autopsies on the causes of neonatal mortality in 2014 showed that the most common causes of neonatal mortality are neonatal sepsis, birth asphyxia, low birth weight, and prematurity-related conditions that occur mostly within the first week of life but, often within the first 24 h following delivery (8, 9).

## Policies and Programs for Newborn Health in Nepal

Nepal has made significant progress in the accessibility of health services in the nation’s rural areas since the endorsement and implementation of specific health policy initiatives in the 1990s. (7). Focused maternal and child health interventions were implemented through various programs such as the National Safe Motherhood Program (NSMP), Community-Based Integrated Management of Childhood Illness (CB-IMCI) (piloted in 1997 and with the nationwide roll-out completed in 2009), bi-annual supplementation of Vitamin A, and the National Program on Immunization. These in combination have resulted in a remarkable reduction in maternal and under-five deaths. In 2004, the Family Health Division of the Ministry of Health (MOH) developed the National Neonatal Health Strategy resulting in newborn health receiving appropriate attention (10). By 2007/08, the Child Health Division had designed the Community-Based Newborn Care Program (CB-NCP), which was first piloted in 10 districts with scaling-up initiated in 2010 (11, 12).

The CB-IMCI was focused on preventative and promotive measures, and treatment for common childhood illnesses, especially neonatal sepsis, measles, malaria, pneumonia, diarrhea, and malnutrition (11). The more recently implemented CB-NCP incorporated seven specific newborn interventions, including, but not limited to, the promotion of essential newborn care, management of neonatal sepsis, Kangaroo Mother Care (KMC) for low birth weight babies, and resuscitating non-breathing babies at birth (12). Female community health volunteers and health workers in rural health care facilities were the service providers in both of the programs. The CB-NCP program was implemented in 41 districts out of 75 up to 2013 (5). However, in 2014, both the CB-NCP and CB-IMCI programs were integrated into the Community-Based Integrated Management of Neonatal and Childhood Illness (CB-IMNCI) that has already been rolled out in 30 districts within the first year of integration (as of July 2015) (11, 13).

Nepal’s NSMP was implemented after the endorsement of the Safe Motherhood Policy in the late 1990s (14). The birth preparedness package, a community-based maternal and newborn health program, was piloted in 2003 with scale-up completed throughout the country in 2008. This program aimed to prepare pregnant mothers for safe birth and complication readiness. NDHS data show that institutional delivery increased from 19 to 36% during 2006–2011 after the implementation of the SBA policy (7, 15, 16). This policy aims to make skilled care at birth available at all Birthing Centers (BCs), Basic Emergency Obstetric and Neonatal Care (BEmONC) sites, as well as Comprehensive Emergency Obstetric and Neonatal Care (CEmONC) sites (16). BCs are the services sites for normal labor and delivery care at rural health facilities, BEmONC sites are for complicated cases such as anticipated assisted deliveries and manual removal of retained placenta, and CEmONC sites are for cesarean deliveries through advanced SBAs and obstetricians (2, 5, 17).

A further development in providing skilled care during delivery was the endorsement of the “National Aama Program,” a maternity incentive scheme. This is a conditional cash transfer program that aims to lower financial barriers for users and aims to facilitate engagement with, and provide access to, ANC visits and institutional delivery.

## Newborn Health Interventions and Their Implementation Status

The seven newborn interventions implemented under the umbrella of child and maternal health programs include skilled care during labor and delivery, antenatal corticosteroids for management of mothers at risk of preterm birth, basic newborn care, managing and resuscitating non-breathing babies at birth (from the Helping Babies Breathe initiative), KMC for stable preterm and low birth weight babies, treatment of neonatal sepsis, and inpatient supportive care for sick and small babies (4).

Table 1 describes the current newborn interventions and their implementation status. The recent coverage of skilled care at birth is about 56%, practices of basic newborn care, for instance, use of chlorhexidine is at 50% and the use of injectable antibiotics (gentamicin) for neonatal sepsis is 32% (17), however, coverage data for other interventions is limited (5, 11).

**Table 1:** **Newborn interventions and their implementation status**.

Programs	Current interventions	Training packages	Main service providers	Service outlets	Suggested packaging of interventions
Maternal health programs	Skilled care at birth Antenatal corticosteroids	Safe motherhood, SBA, Advanced SBA training package	Nurse midwives and doctors	BCs, BEmONCs, and CEmONCs	Skilled care at birth Antenatal corticosteroids Essential newborn care Neonatal resuscitation KMCs
Child health programs	Essential newborn care Neonatal resuscitationKMC service Management of neonatal sepsis Inpatient care for sick and small newborns	CB-IMNCI training package	Paramedical staffs, nurses, and doctors	Peripheral level health facilities and referral hospitals for inpatient care	Management of neonatal sepsis Inpatient care for sick and small newborns

## Challenges in the Implementation of Newborn Health Interventions

Based on their implementation status, challenges can be divided into two groups. First, three interventions: skilled care at birth, basic newborn care, and the management of neonatal sepsis are in a more mature implementation stage. Policy guidelines have been developed, health workers have been trained, a logistics system has been established, and services are available from service outlets. However, achieving high effective coverage is the major barrier with these interventions. The frequently observed health system challenges in these interventions are an inadequate number of skilled service providers or service providers with poor skills, poor coverage and quality of care in available services, and poor and fragile system of logistics procurement and its supply chain management (4, 18–22).

Despite this, the four other interventions for newborns – administration of antenatal corticosteroids for the management of mothers at risk of preterm birth, managing non-breathing babies at birth, providing KMC services for preterm and low birth weight babies, and inpatient care for very small and sick babies – face more critical health system challenges (18). Further development of appropriate policy guidelines for antenatal corticosteroids, KMC services and neonatal inpatient care is required (4, 20, 23, 24). The MOH has not yet issued any guidelines for the management of preterm babies (18). Also, national guidelines for the management of low birth weight babies and national standard inpatient treatment guidelines for severely sick newborns are seriously lacking (18). Proper logistics supply of key items, such as drugs, and bags and masks, is a major barrier in all interventions. The quality of services, and of data use in decision-making and action are further health system challenges. Also, the funding gap in inpatient care services results in inadequate service availability outside the metropolitan areas. All in all, lack of appropriate policies and strategic guidelines, funding gaps, poor infrastructure, and logistics as well as inadequate information are the critical challenges in these newborn health interventions (4, 18).

Table 2 describes newborn interventions and health system challenges for implementation, including possible responses. Ensuring recommended newborn care is challenging in Nepal for a number of reasons (25). Rural BC function is poor due to acute shortages of SBAs as well as the necessary medicines and program commodities. The institutional delivery services provided in the rural BCs are bypassed by women due to perceived poor quality of care and inadequate referral links (26). But overcrowding of the zonal and regional hospitals (26–28), poor infection prevention in BCs and referral hospitals as well as lower or inappropriate use of partographs in the intra-partum period are the greatest challenges for the provision of quality maternal and newborn health services in Nepal (4, 8). Compounding challenges are the socio-cultural differences and practices that may create difficulties in regard to postnatal care (PNC). In addition, adopting the current protocol for PNC visits to health care facilities is challenging in areas where postpartum mothers do not leave their homes until 10 days after the delivery (7). More than half of all newborn deaths reportedly occur due to perinatal complications (29), but in the given context, quality intra-partum care services is not being properly delivered (27). Furthermore, very few tertiary hospitals offer KMC services because of inadequately skilled health care providers and space in those facilities (30, 31). Inadequate BEmONC and CEmONC sites in remote districts have resulted in poor coverage of services as well as poor practices in management of complications (4). The poor infrastructure development of Neonatal Intensive Care Units (NICU), combined with limited access to these services, are the significant health system issues in the delivery of effective maternal and newborn services (4). While the MOH has implemented free delivery care services in public hospitals, for various reasons, people reportedly have to pay for inpatient newborn care services in all hospitals (32).

**Table 2:** **Newborn interventions and health system implementation challenges**.

Categories of newborns	Newborn health interventions (time of care)	Service outlets	Service providers (currently available services)	Policies, strategies, programs, and training packages	Health system challenges	Possible actions
Care for all newborns	Skilled care during labor and delivery (during labor and delivery)	BCs, BEmONCs, and CEmONCs	SBAs (safe and clean birth, use of partograph) doctors (assisted delivery) Advanced SBA and obstetricians (c-section)	Policies and program on safe motherhood and skilled birth attendance, maternity incentive scheme program, CB-IMNCI program, and SBA package	Inequity of institutional delivery services especially among poor, disadvantaged and remote communities Quality of care during labor and delivery Inadequate supply of medical supplies Low coverage of BEmONC and CEmONC services Poor data quality, insufficient mentoring and onsite coaching support Low or no use of partograph Inadequate number of SBA and their unequal distribution	Develop and implement the remote area strategy Use quality of care guideline to ensure optimal standard of care Ensure quality of care audit Perinatal and neonatal death audit Train adequate number of SBAs and deployed in hard-to-reach health facilities Male involvement in support pregnancy and delivery care Appropriate logistics supplies Technical support visits, onsite coaching and mentoring Educational campaigns and mobile apps on importance of skilled care at birth
	Antenatal corticosteroids for management of mothers at risk of preterm birth (during labor)	Specialized hospitals	Obstetricians (antenatal corticosteroids for fetal lung maturation)	National medical standards	Lacking policy guideline to implement in BEmONC Prescription authority is not to SBAs Indicators lacking in routine information system Inadequate family and community awareness on preterm labor	Ensure service available at BEmONC sites and expand such services in remote districts Task shifting to SBAs Include in routine information system Community mobilization on complication of preterm labor Include antenatal corticosteroids in essential drugs list
	Basic newborn care (immediate after labor)	Health facilities, communities, and home	SBAs, paramedics, and doctors (cleanliness, thermal care-drying and wrapping, skin-to-skin contact, and delaying bathing, cord care with chlorhexidine, and immediate breastfeeding)	CB-IMNCI program, SBA package	Poor adherences to essential newborn care standards Low programmatic priority of birth prepared package Low coverage of ENCs services Inadequate competency of service providers on ENCs Poor Data quality of ENCs Poor community awareness Poor supply chain management	Implement behavior change communication activities to reduce harmful practices Onsite coaching and mentoring to improve skills of service providers Conduct program performance reviews meetings Improve family and community practices through mass media mobilization Increase male involvement in basic newborn care Ensure proper supplies especially chlorhexidine gel
Care of all newborns who are at risks –small and sick newborns	Managing non-breathing babies at birth-neonatal resuscitation (immediate after labor)	BCs, BEmONC, and CEmONC	SBAs (neonatal resuscitation using bag and mask)	CB-IMNCI program, SBA training package	Poor competency of SBAs to manage the birth asphyxia Inadequate supply of bag and mask SBAs reluctance to handle birth asphyxia conditions Data are not captured in health information system Inadequate harmonization in pre-service curricula	Onsite coaching and mentoring of SBAs Ensure the availability of resuscitation commodities Ensure newborn resuscitation service data in health information management system (HMIS) Create awareness on complication of birth asphyxia Include pre-service training packages and train medical and nursing students
	KMC for stable preterm and low birth weight babies (postnatal)	BCs, BEmONC, and CEmONC	SBAs (kangaroo mother care for stable newborn who are preterm and low birth weights	SBA package, CB-IMNCI program	Lack of standard national protocol Poor supply of logistics to perform KMC Poor community perception of KMC Inadequate space in HFs for KMC services on ENCs Lack of postnatal care guideline for newborn born at home Inadequate information on KMC services Poor skills of health workers	Promote KMC practices at health facilities Ensure postnatal care of all newborns Establish KMC corners in each birthing centers Ensure KMC service data in HMIS Mobilize community and increase on benefits of KMC Increase the demand of KMC services Involve male in KMC services Train health services providers
	Treatment of neonatal sepsis (0-59 days)	Peripheral level health facilities and community	Paramedics, SBAs, and doctors (injectable antibiotics-gentamicin)	CB-IMNCI program	Poor adherence of national protocol especially in private sector Lack of information services received from private sector Poor skills and competency of health workers Weak referral mechanism Delay procurement and poor supply chain management Poor quality of services Poor care seeking behavior of communities	Mentor health workers provide technical skills to health workers Develop appropriate strategy to engage private sector Ensure the data quality by performance review meeting and monitoring Include treatment protocol in pre-service training course and involve academias Ensure year round availability of essential drugs to treat neonatal sepsis Develop contextual IEC materials to educate communities
	Inpatient supportive care for sick and small babies (0–59 days)	Secondary and tertiary referral hospitals, special newborn care unit	Doctors, pediatricians (intravenous fluids, alternative feeding supports, and oxygen support)	Guideline is yet to be developed	Lack of standard protocols Funding gaps in inpatient care Unavailability of Inpatient care services for newborn at inpatient unit of all district hospital Poor supply of essential commodities for inpatient care for newborn Poor documentation of service statistics Lack of neonatal nurse and other skilled human resources Inadequate budget for upgrading HFs to Special care Neonatal Unit (SCNU) and establishing Neonatal Intensive Care Unit (NICU) services at referral hospitals Weak referral system due to transportation and financial barriers	Prepare and implement inpatient care guideline for very small and severely sick neonates Develop NICU in referral hospital and special newborn care unit at district hospitals Provide free newborn care for all referred newborns who need inpatient care services including transportation cost Ensure funds for NICU and SCNU development and operation Use quality of care guideline Develop local transportation system Develop innovative financial mechanism (voucher system, insurance or incentive system)

In rural areas, some BCs face inadequacy and incompetence among SBAs to provide neonatal resuscitation services (33). Also, an unequal distribution of care providers has resulted in lower rates of institutional delivery, although Nepal had aimed to achieve a rate of 60% institutional delivery by SBAs by 2015 (4). In many maternal and newborn health training programs and packages and in pre- and in-service training, newborn components and skills are not emphasized. Vertical program planning and implementation at different levels, inadequate data use within program management, poor supervision, and program monitoring are some of the health system inadequacies in newborn health care (5).

In recent years, provisions of funding and interest have increased and some interventions have been mainstreamed in maternal and child health programs. The inclusion of the newborn components in NSMP was also initiated, but newborn health interventions were either diluted in the safe motherhood program or have received less attention during program implementation (5). Unfortunately, strategies, guidelines, and protocols on safe motherhood and newborn health are not aligned (4). Fragmentation of newborn health interventions from maternal health programs also clearly shows the difficulties in service delivery and has further compounded the challenges. For example, most of the interventions of the CB-NCP have been merged with IMCI but they ought to be integrated with NSMP as SBAs are the primary service providers of skilled care at birth (11, 14) (Table 1).

## Conclusion

The health of the newborn is an important component of maternal care. Therefore the strategic integration of all interventions is strongly recommended, not only at the implementation level but also at the policy level. Equal access to and utilization of quality antepartum, intra-partum, postpartum, and inpatient care are essential to save the lives of newborns. The crucial health system challenges such as poor and inadequate skills in preventing, identifying, and managing newborn complications, inadequate infrastructure, unfavorable working environments and poor supply of essential newborn commodities, ineffective clinical supervision, and mentorship for service providers all need to be addressed and improved to ensure effective coverage of newborn interventions. Health facility-based interventions should focus on quality of care in newborn services; in particular, inpatient care services for sick and low birth weight newborns should be made available in each district hospital. Investment and interventions should be targeted at remote and hard-to-reach rural health facilities. The combination of these interventions will contribute to further lowering Nepal’s NMR.

## Author Contributions

All authors listed, have made substantial, direct and intellectual contribution to the work, and approved it for publication.

## Conflict of Interest Statement

The authors declare that the research was conducted in the absence of any commercial or financial relationships that could be construed as a potential conflict of interest.

## References

[1] Neupane S Doku DT Neonatal mortality in Nepal: a multilevel analysis of a nationally representative. J Epidemiol Glob Health (2014) 4(3):213–22. 10.1016/j.jegh.2014.02.001 25107657 PMC7333823

[2] Paudel D Shrestha IB Siebeck M Rehfuess EA. Neonatal health in Nepal: analysis of absolute and relative inequalities and impact of current efforts to reduce neonatal mortality. BMC Public Health (2013) 13(1):1239. 10.1186/1471-2458-13-1239 24373558 PMC3890515

[3] Paudel D Thapa A Shedain P Paudel B Trends and Determinants of Neonatal Mortality in Nepal: Further Analysis of the Nepal Demographic and Health Surveys, 2001-2011. Kathmandu; Calverton, MD: Nepal Ministry of Health and Population, New ERA; ICF International (2013).

[4] MoHP; UNICEF; Save the Children; WHO; USAID. Every Newborn Action Plan-Report on National Consultative Workshop – Nepal (Draft). Kathmandu: Ministry of Health and Population, UNICEF, Save the Children, WHO and USAID (2013).

[5] Child Health Division; Family Health Division; Save the Children. A Synthesis of Recent Studies on Maternal and Newborn Survival Interventions in Nepal. Kathmandu: Child Health Division and Family Health Division, Ministry of Health and Population (2014).

[6] Pradhan YV Upreti SR Kc NP Ashish KC Khadka N Syed U Newborn survival in Nepal: a decade of change and future implications. Health Policy Plan (2012) 27(Suppl 3):iii57–71. 10.1093/heapol/czs052 22692416

[7] Ministry of Health and Population (MOHP) [Nepal]; New ERA; ICF International Inc. Nepal Demographic and Health Survey 2011. Kathmandu; Calverton, MD: Ministry of Health and Population, New ERA; ICF International (2012).

[8] Ministry of Health and Population; USAID; IRHDTC Nepal. A Report on Verbal Autopsy to Ascertain the Causes of Neonatal Deaths in Nepal. Kathmandu: Ministry of Health and Population, USAID, IRHDTC Nepal (2014).

[9] Khanal S Gc VS Dawson P Houston R. Verbal autopsy to ascertain causes of neonatal deaths in a community setting: a study from Morang, Nepal. JNMA J Nepal Med Assoc (2011) 51(181):21–7. 22335091

[10] Khadka N Moore J Vickery J Nepal’s Neonatal Health Strategy: A Policy Framework for Program Development. Maryland: JPHIEGO (2003).

[11] Child Health Division. CB-IMNCI Report 2015-Draft. Kathmandu Nepal: Child Health Division (2015).

[12] Child Health Division. Assessment of the Community Based Newborn Care Package. Kathmandu: Child Health Division, Ministry of Health and Population (2012).

[13] Bhandari N Mazumder S Taneja S Sommerfelt H Strand TA Effect of implementation of integrated management of neonatal and childhood illness (IMNCI) programme on neonatal and infant mortality: cluster randomised controlled trial. BMJ (2012) 344:e1634 10.1136/bmj.e1634 22438367 PMC3309879

[14] Bhandari A Pradhan YN Kc NP Upreti SR Thapa K Sharma G State of maternal, newborn and child health programmes in Nepal: what may a continuum of care model mean for more effective and efficient service delivery? J Nepal Health Res Counc (2011) 9(2):92–100. 22929837

[15] Ministry of Health and Population (MOHP) [Nepal]; New ERA; ICF International Inc. Nepal Demographic Health Survey 2006. Kathmandu; Caverton, MD: Ministry of Health and Population, New ERA; ICF International (2007).

[16] Family Health Division. National Policy on Skilled Birth Attendants. Kathmandu: Family Health Division, Ministry of Health and Population (2006).

[17] Ministry of Health and Population. Annual Report. Nepal: Management Division, Ministry of Health and Population (2012/13).

[18] Dickson KE Kinney MV Moxon SG Ashton J Zaka N Simen-Kapeu A Scaling up quality care for mothers and newborns around the time of birth: an overview of methods and analyses of intervention-specific bottlenecks and solutions. BMC Pregnancy Childbirth (2015) 15(Suppl 2):S1. 10.1186/1471-2393-15-S2-S1 26390820 PMC4578819

[19] Health for Life (H4L); ICF International Inc.; New ERA. Monitoring the Progress of the Birthing Centers in Nepal. Kathmandu: Health for Life (H4L), ICF International Inc.,& New ERA (2014).

[20] Moxon SG Lawn JE Dickson KE Simen-Kapeu A Gupta G Deorari A Inpatient care of small and sick newborns: a multi-country analysis of health system bottlenecks and potential solutions. BMC Pregnancy Childbirth (2015) 15(Suppl 2):S7. 10.1186/1471-2393-15-S2-S7 26391335 PMC4577807

[21] Simen-Kapeu A Seale AC Wall S Nyange C Qazi SA Moxon SG Treatment of neonatal infections: a multi-country analysis of health system bottlenecks and potential solutions. BMC Pregnancy Childbirth (2015) 15(Suppl 2):S6. 10.1186/1471-2393-15-S2-S6 26391217 PMC4578441

[22] Sharma G Mathai M Dickson KE Weeks A Hofmeyr GJ Lavender T Quality care during labour and birth: a multi-country analysis of health system bottlenecks and potential solutions. BMC Pregnancy Childbirth (2015) 15(Suppl 2):S2. 10.1186/1471-2393-15-S2-S2 26390886 PMC4577867

[23] Vesel L Bergh A-M Kerber KJ Valsangkar B Mazia G Moxon SG Kangaroo mother care: a multi-country analysis of health system bottlenecks and potential solutions. BMC Pregnancy Childbirth (2015) 15(Suppl 2):S5. 10.1186/1471-2393-15-S2-S5 26391115 PMC4577801

[24] Liu G Segrè J Gülmezoglu AM Mathai M Smith JM Hermida J Antenatal corticosteroids for management of preterm birth: a multi-country analysis of health system bottlenecks and potential solutions. BMC Pregnancy Childbirth (2015) 15(Suppl 2):S3. 10.1186/1471-2393-15-S2-S3 26390927 PMC4577756

[25] Navaneetham K Dharmalingam A Demography and development: preliminary interpretations of the 2011 census. Econ Polit Wkly (2013) 46(16):13.

[26] Karkee R Binns CW Lee AH. Determinants of facility delivery after implementation of safer mother programme in Nepal: a prospective cohort study. BMC Pregnancy Childbirth (2013) 13(1):193. 10.1186/1471-2393-13-193 24139382 PMC3816171

[27] Karkee R Lee AH Binns CW. Bypassing birth centres for childbirth: an analysis of data from a community-based prospective cohort study in Nepal. Health Policy Plan (2015) 30(1):1–7. 10.1093/heapol/czt090 24270520

[28] Karkee R Lee AH Pokharel PK. Women’s perception of quality of maternity services: a longitudinal survey in Nepal. BMC Pregnancy Childbirth (2014) 14(1):45. 10.1186/1471-2393-14-45 24456544 PMC3902186

[29] Bhutta ZA Das JK Bahl R Lawn JE Salam RA Paul VK Can available interventions end preventable deaths in mothers, newborn babies, and stillbirths, and at what cost? Lancet (2014) 384(9940):347–70. 10.1016/S0140-6736(14)60792-3 24853604

[30] Subedi K Aryal DR Gurubacharya SM Kangaroo mother care for low birth weight babies: a prospective observational study. J Nepal Paediatr Soc (2009) 29(1):6–9. 10.3126/jnps.v29i1.1593

[31] Acharya N Singh RR Bhatta NK Poudel P Randomized control trial of kangaroo mother care in low birth weight babies at a tertiary level hospital. J Nepal Paediatr Soc (2014) 34(1):18–23. 10.3126/jnps.v34i1.8960

[32] Upreti S Baral S Lamichhane P Khanal M Tiwari S Tandan M Rapid Assessment of the Demand Side Financing Schemes: Aama and 4ANC Programmes (The Seventh Rapid Assessment). Kathmandu: Ministry of Health and Population, Nepal Health Sector Support Programme and Health Research and Social Development Forum (2013).

[33] Enweronu-Laryea C Dickson KE Moxon SG Simen-Kapeu A Nyange C Niermeyer S Basic newborn care and neonatal resuscitation: a multi-country analysis of health system bottlenecks and potential solutions. BMC Pregnancy Childbirth (2015) 15(Suppl 2):S4. 10.1186/1471-2393-15-S2-S4 26391000 PMC4577863

